# Assessing the association of physical distancing to avoid COVID-19 with health-related quality of life in immunocompromised adolescents: results from the cross-sectional observational EAGLE study

**DOI:** 10.3389/fped.2026.1771834

**Published:** 2026-05-28

**Authors:** Paul Williams, Timothy A. Herring, Renata T. C. Yokota, Sudhir Venkatesan, Klas Bergenheim, Johan L. Severens, Philip A. Powell, James C. Marcus, Stephanie Philpott, Sara Bestea, Jeffrey M. Rohay, Tiago Maia, Sylvia Taylor, Marieke Krol, James W. Varni

**Affiliations:** 1 Medical Evidence, BioPharmaceuticals Medical, AstraZeneca, Gothenburg, Sweden; 2 Medical Evidence, Vaccines & Immune Therapies, BioPharmaceuticals Medical, AstraZeneca, Wilmington, DE, United States; 3 P95 Clinical and Epidemiology Services, Leuven, Belgium; 4 BPM Evidence Statistics, BioPharmaceuticals Medical, AstraZeneca, Cambridge, United Kingdom; 5 Health Economics & Payer Evidence, BioPharmaceuticals Market Access, AstraZeneca, Gothenburg, Sweden; 6 Severens HTA Consultancy, Heemskerk, Netherlands; 7 Philip A Powell Consulting, Sheffield, United Kingdom; 8 IQVIA, Washington, DC, United States; 9 IQVIA, Reading, United Kingdom; 10 IQVIA, Madrid, Spain; 11 IQVIA, New York, NY, United States; 12 IQVIA, Porto Salvo, Portugal; 13 Medical Evidence, Vaccines & Immune Therapies, BioPharmaceuticals Medical, AstraZeneca, Cambridge, United Kingdom; 14 IQVIA, Amsterdam, Netherlands; 15 Texas A&M University, College Station, TX, United States

**Keywords:** adolescent, COVID-19, health-related quality of life, health-state utilities, immunocompromised, physical distancing, SARS-CoV-2, social isolation

## Abstract

**Objective:**

We describe physical distancing behaviors to avoid coronavirus disease 2019 (COVID-19) and their associations with health-related quality of life (HRQoL) and related outcomes, among immunocompromised adolescents (aged 13–17 years).

**Methods:**

EAGLE was a cross-sectional, observational study of immunocompromised adults, adolescents, and children in the United States and United Kingdom. Adolescents and their caregivers were enrolled between February and June 2023 and completed a web-based survey that was designed to capture the following outcomes: physical distancing behaviors in the past 4 weeks, measured using the Physical Distancing Scale for COVID-19 Avoidance (PDS-C19®); HRQoL (Pediatric Quality of Life Inventory™ [PedsQL™] Generic Core Scales); loneliness (Direct Measure of Loneliness [DMOL] scale); health-state utility (EQ-5D-5L); mental health (Hospital Anxiety and Depression Scale; HADS®); and school and activity impairment (Work Productivity and Activity Impairment plus Classroom Impairment Questions: Specific Health Problem questionnaire; WPAI-CIQ:SHP).

**Results:**

Among 405 immunocompromised adolescents, the PDS-C19 mean T-score was 49.1, indicative of moderate physical distancing intensity. Most participants reported moderate (60.1%) or high/very high (16.3%) physical distancing intensity; fewer reported low (10.0%) or very low (13.7%) physical distancing intensity. The PedsQL™ Generic Core Scales mean total score was 58.0 (scale range: 0–100, where higher scores indicate better HRQoL). Most outcomes moderately correlated with PDS-C19 (|*r*| = 0.4–0.5), with stronger correlations (|*r*|>0.6) shown for WPAI-CIQ:SHP activity impairment, school presenteeism, and overall school impairment. Linear regression models adjusting for confounders showed similar associations.

**Conclusions:**

Two years after national lockdowns ended, most immunocompromised adolescents practiced moderate-to-high intensities of physical distancing to avoid COVID-19. Higher intensities of physical distancing were associated with worse HRQoL and greater school and activity impairment, emphasizing the prolonged burden of COVID-19 avoidance in this population.

## 1 Introduction

Immunocompromised individuals may have suboptimal responses to coronavirus disease 2019 (COVID-19) vaccines (1–4) and are at increased risk of severe COVID-19 outcomes despite vaccination (5–7). Many health authorities had lifted COVID-19–related societal restrictions by 2022 (8, 9), and in May 2023, the World Health Organization declared that COVID-19 was no longer an international public health emergency (10). However, given the increased risks in the immunocompromised population, post-pandemic national guidance from health authorities in the United States (US) and the United Kingdom (UK) advised that immunocompromised individuals may consider continuing to practice individual-level, risk-reducing behaviors to protect against COVID-19, including physical distancing such as avoiding or reducing time spent in crowded places and increasing space between oneself and others (11, 12).

Although important for the prevention of COVID-19, the detrimental impact of physical distancing to avoid COVID-19 on the lives of individuals during lockdown periods has been demonstrated in several populations. Among immunocompromised adults, physical distancing was demonstrated to negatively impact various aspects of physical and mental health (13–17). Among nonimmunocompromised children and adolescents, studies conducted across several regions during the COVID-19 pandemic have documented associations between physical distancing and numerous parameters. These include worse physical health (e.g., weight gain and decreased physical activity), worse mental health (e.g., depression and anxiety), decreased life satisfaction and mental health correlates (e.g., increased screentime) (18–20), decreased vaccination coverage and uptake of routine childhood vaccinations (19), and reduced learning and school performance due to school closures (21).

Very limited published data exist describing the intensity of physical distancing behaviors practiced by immunocompromised adolescents, including those to avoid COVID-19, and the impact such behaviors have on their daily lives (22, 23). EAGLE was a US- and UK-based observational, cross-sectional study that assessed the intensity of physical distancing behaviors to avoid COVID-19 and its associations with health-related quality of life (HRQoL) and related measures among immunocompromised adults, adolescents, children, and the caregivers of adolescents and children (24–26). Here, we present physical distancing behaviors adopted by immunocompromised adolescents (aged 13–17 years) to avoid COVID-19, and their association with HRQoL and reported outcomes.

## 2 Methods

### 2.1 Study design

EAGLE was a noninterventional, observational, cross-sectional study of immunocompromised adults (aged ≥18 years), adolescents (aged 13–17 years), children (aged 6 months–12 years), and caregivers of those adolescents and children (AstraZeneca study code: D8850R00013). Caregivers were defined as parents or legal guardians aged ≥18 years and who always, or at least sometimes, lived with the child or adolescent (24). The study was performed in the US and the UK in the post-lockdown period between December 15, 2022, and June 2, 2023. Nonimmunocompromised individuals were also included for informal benchmarking, not formal comparison. A summary of the study design and protocol of EAGLE has been reported previously (24). Patients were involved in the design of the study protocol; their role has been described previously (24).

The study comprised a survey that was administered once, via an electronic, secure, web-based platform, with self- and/or caregiver-reported responses. The survey consisted of questions in English (US/UK) and Spanish (US), which were designed to be completed in approximately 30–45 min in total. For adolescents, the survey comprised sections for self-completion (adolescent outcomes) and completion by their caregivers (demographic and clinical characteristics of adolescents, as well as caregiver-specific outcomes). Adolescents self-completed their sections first, and so the total time for adolescents to complete the survey was expected to be less than 15 min.

The results for immunocompromised adults in the EAGLE study have been reported previously (25, 26). Participant recruitment and data collection for immunocompromised adolescents and their caregivers took place in the post-lockdown period between February and June 2023. Caregiver-specific outcomes are not reported herein.

### 2.2 Study ethics

The study was performed in accordance with ethical principles consistent with the Declaration of Helsinki, Good Pharmacoepidemiology Practices, and the applicable legislation and/or regulations on noninterventional studies and/or observational studies. The study received institutional review board (IRB) approval in the US [WCG IRB. Letter of approval of EAGLE Study Protocol v1.0 and related material. IRB tracking number: 20226100. November 15, 2022]. IRB approval of the study was not required in the UK as participants were recruited outside of the National Health Service. All adolescent participants provided assent, and their respective caregivers (i.e., a parent or legal guardian) provided informed consent, electronically, before entering the study.

### 2.3 Participants and recruitment

Adolescents (aged 13–17 years) residing in the US and the UK were eligible if their caregivers (aged ≥18 years) reported that they had at least one of the following moderate-to-severe immunocompromising conditions or had received immunosuppressive treatments within 2 months prior to enrollment: hematologic malignancies, active treatment for solid tumors, solid organ or stem cell transplant, end-stage kidney disease, primary immunodeficiency disorders, receipt of immunosuppressant treatment, uncontrolled human immunodeficiency virus infection, or other immunocompromising condition. Immunocompromised conditions were defined based on the UK government and US Centers for Disease Control and Prevention guidelines (27, 28). Individuals without immunocompromise but with a contraindication to COVID-19 vaccination (e.g., an allergy to COVID-19 vaccine components) were also considered as immunocompromised for this study because they may remain susceptible to severe COVID-19 outcomes. Nonimmunocompromised adolescents were included if their caregiver reported that they did not have any of the specified immunocompromising conditions or had not received immunocompromising treatment since January 2020. Additional eligibility criteria for adolescent study participants are listed in the Supplementary Material.

Participants were identified and recruited by Global Perspectives (an IQVIA company), via multiple direct-to-patient channels of recruitment, including patient panels and networks, clinician referral networks, patient advocacy groups, and social media outreach. Interested and eligible participants were emailed the survey link and informed that completion was required within 1 week of providing consent.

Caregivers reported the immunocompromised status of adolescent participants; however, to aid in validating such self-reports, confirmation of diagnosis, in the form of medical documentation of immunocompromised status or receipt of immunocompromising treatment, was requested from a randomly selected sample of approximately 25% of study participants across all age groups and immunocompromising categories.

Caregivers of adolescent participants received an honorarium after completing their survey to compensate them for their time, and those who provided confirmation of diagnosis received an additional honorarium. The value of the honoraria was based on fair market value as per local country guidance.

### 2.4 Reported outcomes

The survey captured physical distancing behaviors to avoid COVID-19, and HRQoL measures and health-state utilities. The study outcomes included were developmentally appropriate for adolescent self-report, with caregiver assistance for the adolescent self-report sections provided only when necessary, as described in the survey instructions.

Physical distancing behaviors to avoid COVID-19 were assessed using the Physical Distancing Scale for COVID-19 Avoidance (PDS-C19®), a novel, validated, self- and observer-reported instrument for both immunocompromised and nonimmunocompromised individuals (24, 29). The PDS-C19 includes seven items, each on a five-point Likert scale (with response options of: “Never,” “Rarely,” “Sometimes,” “Often,” and “Always”), capturing intensity of physical distancing behavior to avoid COVID-19 over the past 4 weeks. Item scores were summed and converted to a T-score (mean [standard deviation; SD] = 50 [10]) using a validated scoring map. T-scores were categorized into four SD-based intensity levels (<–2; −2 to < –1; −1 to <1; ≥1): very low (<30), low (30–39.99), moderate (40–59.99), and high/very high (≥60).

HRQoL was measured in adolescents using the Pediatric Quality of Life Inventory™ (PedsQL™) Generic Core Scales (30) and the Direct Measure of Loneliness (DMOL) scale (31). Health-state utility was assessed using the EQ-5D-5L questionnaire (32), with utility scores derived from UK (33) and US (34) value sets. Mental health was assessed using the Hospital Anxiety and Depression Scale (HADS®) (35). School and activity impairment were assessed using the Work Productivity and Activity Impairment plus Classroom Impairment Questions: Specific Health Problem questionnaire (WPAI-CIQ:SHP) (36). All adolescents completed the WPAI-CIQ:SHP activity impairment domain; work-related items were omitted, and only those who attended classes in an academic setting (middle school, high school, college, graduate school, additional course work, etc.) completed the school absenteeism and school presenteeism domains, which are used to calculate overall school impairment.

All instruments were scored as per their respective license-holders' scoring manuals. Additional details describing the instruments, including scoring and interpretation, are provided in the Supplementary Materials.

### 2.5 Statistical analysis

As this was a descriptive study, no formal sample size or power calculations were performed. However, a feasibility assessment was conducted to inform the sampling of key groups and achieve representation across a variety of immunocompromising conditions and COVID-19 physical distancing behavior intensities; additional details are provided in the published study design and protocol (24).

Initially and prior to conducting the descriptive analyses presented here, developmental work (in the form of focus groups) for the PDS-C19 was conducted, as well as psychometric validation in a randomly selected subset (*n* = 1,059) of study respondents across all age groups and immunocompromised status, including 152 adolescents. The results of these previous works have been reported elsewhere (29, 37). Unless otherwise stated, all analyses presented here were conducted on the associations analysis set, which included all eligible adolescents who fully completed the survey in the full analysis set except the 113 from the full analysis set who were selected for PDS-C19 psychometric validation.

Descriptive statistics were used to summarize participant characteristics, PDS-C19 physical distancing intensity groups (very low, low, moderate, high/very high), and domain-level scores of reported outcomes. Missing data were tabulated and excluded from the proportion calculations.

For immunocompromised adolescents, associations between PDS-C19 scores and reported outcomes were estimated using Pearson correlation. In this framework, correlation magnitudes are categorized as: negligible (|*r*|<0.2), low (|*r*|≥0.2 to <0.5), medium (|*r*|≥0.5 to <0.8), and high (|*r*|≥0.8). In social science research, |*r*| = 0.2 represents a minimum threshold for “practical” significance (38), while |*r*| = 0.5 represents the largest magnitude effect typically observed between constructs (39).

Separate linear regression models were fitted for each outcome, with the PDS-C19 score as the primary predictor of interest. Models were adjusted for potential confounders selected across three domains: other available risk mitigation behavior variables (mask wearing, others isolating to protect the participant, number of COVID-19 vaccine doses received), COVID-19–related worry perceptions (perception of infection risk, perceived potential severity of COVID-19, knowledge of family/friend deaths due to COVID-19), and demographic/clinical variables (sex, ethnicity, household income, immunocompromising category, and caregiver immunocompromised status). Results are reported as unstandardized regression coefficients (*B*) with standard errors (SEs).

A series of four structural equation modeling (SEM)-based path analyses were conducted to explore the direct and indirect relationships between PDS-C19 and the following conceptually grouped outcome domains: HRQoL, health-state utility, other outcomes for the total sample of participants, and other outcomes for those participants still in school. Covariates included in SEMs were consistent with those in regression models, except for the number of COVID-19 vaccine doses received, ethnicity, and immunocompromising category, which were excluded as exogenous variables. Risk mitigation behaviors (PDS-C19, mask wearing, others isolating) were modeled as first-layer endogenous variables; DMOL (loneliness) as the second layer; and grouped outcome domains as the final layer. A filled path diagram for HRQoL outcomes, as an exemplar of the SEM models, is provided in Supplementary Figure S1. SEM results are reported in tables as unstandardized regression coefficients (*B*) and standardized coefficients (*β*), both with SEs. The SEM results reported here are limited to those involving the association between PDS-C19 and HRQoL and other reported measures.

Statistical analyses were performed using SAS® version 9.4 (SAS Institute Inc., Cary, NC, USA). SEMs were fit via Mplus version 8.8 (Muthen & Muthen, Los Angeles, CA, USA) using a weighted least squares mean and variance adjusted estimator. For inferential analyses (SEM and regressions), *P*-values were not adjusted for multiplicity across analyses, and reported *P*-values <0.05 are nominal and must only be interpreted as being nominally statistically significant in the context of the number of analyses conducted.

## 3 Results

### 3.1 Participants

In total, 634 adolescents were enrolled in the EAGLE study between February 3 and June 2, 2023 and completed the full survey (100% response). Of these, 491 adolescents were immunocompromised and were included in either the associations analysis set (*n* = 405 [US, *n* = 260; UK, *n* = 145]) or the PDS-C19 psychometric validation set (*n* = 86) (Supplementary Figure S2). Furthermore, 143 adolescents were nonimmunocompromised and were included in either the associations analysis set (*n* = 116) or the PDS-C19 psychometric validation set (*n* = 27) (Supplementary Figure S2).

Of the 405 immunocompromised adolescents included in the associations analysis set, 51.4% (*n* = 208) were female and most were White (in the US: 63.1%, *n* = 164; in the UK: 72.4%, *n* = 105) (Table 1). Most immunocompromised adolescents had one immunocompromising condition (81.5%, *n* = 330), most commonly primary immunodeficiency disorder (25.4%, *n* = 103), solid organ or stem cell transplant (21.5%, *n* = 87), and immunosuppressant treatment (10.4%, *n* = 42); 24.7% (*n* = 100) had other immunocompromising disorders.

**Table 1:** Demographic and clinical characteristics of the immunocompromised adolescent participants of the EAGLE study: US and UK, February to June 2023.

**Characteristic, *n* (%)**	**US**	**UK**	**Total**
	**(*n* = 260)**	**(*n* = 145)**	**(*N* = 405)**
Immunocompromising category^a^			
Primary immunodeficiency disorder	73 (28.1)	30 (20.7)	103 (25.4)
Solid organ or stem cell transplant	28 (10.8)	59 (40.7)	87 (21.5)
Immunosuppressant treatment	24 (9.2)	18 (12.4)	42 (10.4)
Blood cancer	11 (4.2)	13 (9.0)	24 (5.9)
Contraindicated to COVID-19 vaccination	20 (7.7)	1 (0.7)	21 (5.2)
End-stage kidney disease	9 (3.5)	4 (2.8)	13 (3.2)
On active treatment for solid tumors	6 (2.3)	2 (1.4)	8 (2.0)
Uncontrolled HIV infection	7 (2.7)	0	7 (1.7)
Other disorder	82 (31.5)	18 (12.4)	100 (24.7)
Sex			
Female	145 (55.8)	63 (43.4)	208 (51.4)
Race (US; *n* = 260)^b^			
White	164 (63.1)	–	–
Black or African American	59 (22.7)	–	–
Hispanic	21 (8.1)	–	–
Asian	18 (6.9)	–	–
American Indian or Alaska Native	6 (2.3)	–	–
Native Hawaiian or other Pacific Islander	3 (1.2)	–	–
Other	2 (0.8)	–	–
Prefer not to say	6 (2.3)	–	–
Race (UK; *n* = 145)^b^			
White	–	105 (72.4)	–
Black, Black British, Caribbean, or African	–	17 (11.7)	–
Mixed or multiple ethnic groups	–	20 (13.8)	–
Asian or Asian British	–	4 (2.8)	–
Prefer not to say	–	1 (0.7)	–
Adults (aged ≥18 years) living in household			
0	8 (3.1)	1 (0.7)	9 (2.2)
1	32 (12.3)	10 (6.9)	42 (10.4)
2	177 (68.1)	104 (71.7)	281 (69.4)
≥3	43 (16.5)	30 (20.7)	73 (18.0)
Children (aged <18 years) living in household			
0	2 (0.8)	0	2 (0.5)
1	105 (40.4)	27 (18.6)	132 (32.6)
2	108 (41.5)	72 (49.7)	180 (44.4)
≥3	45 (17.3)	46 (31.7)	91 (22.5)
Attending classes in an academic setting^c^			
Yes	214 (82.3)	125 (86.2)	339 (83.7)
No	46 (17.7)	20 (13.8)	66 (16.3)
Ever tested positive for SARS-CoV-2			
Yes	44 (16.9)	21 (14.5)	65 (16.0)
No	195 (75.0)	120 (82.8)	315 (77.8)
Unknown	21 (8.1)	4 (2.8)	25 (6.2)
Hospitalized due to SARS-CoV-2			
Yes	11 (4.2)	13 (9.0)	24 (5.9)
No	249 (95.8)	132 (91.0)	381 (94.1)
Number of SARS-CoV-2 vaccines received			
0	102 (39.2)	44 (30.3)	146 (36.0)
1	44 (16.9)	3 (2.1)	47 (11.6)
2	67 (25.8)	17 (11.7)	84 (20.7)
3	36 (13.8)	26 (17.9)	62 (15.3)
≥4	11 (4.2)	55 (37.9)	66 (16.3)

Data are from the associations analysis set (*N* = 405), comprising the full analysis set (*n* = 491) excluding the psychometric evaluation set. En dash (–) indicates not applicable.

a Immunocompromising category was derived by mutually exclusive categorization; patients who were not included in any one of the eight predefined categories were placed in the “Other disorder” category.

b Participants may have selected more than one.

c Middle school, high school, college, graduate school, additional course work, etc.

COVID-19, coronavirus disease 2019; HIV, human immunodeficiency virus; SARS-CoV-2, severe acute respiratory syndrome coronavirus 2; UK, United Kingdom; US, United States.

Most of the immunocompromised adolescents had been told by a healthcare provider that they were immunocompromised (77.3%, *n* = 313). Of 493 immunocompromised adolescents in the full analysis set, 31 (6.3%) were randomly selected to provide confirmation of diagnosis documentation. Of these, 17 (54.8%) provided confirmation of diagnosis evidence, and 15 of those 17 (88.2%) had their evidence validated. The remaining two adolescents were found to have invalid confirmation of diagnosis.

Most immunocompromised adolescents reported never testing positive for severe acute respiratory syndrome coronavirus 2 (SARS-CoV-2; 77.8%, *n* = 315) infection, and most reported never being hospitalized due to SARS-CoV-2 infection (94.1%, *n* = 381). Most reported that they had received at least one dose of SARS-CoV-2 vaccine (64.0%, *n* = 259).

Select baseline characteristics for nonimmunocompromised adolescents are reported in Supplementary Table S1.

### 3.2 COVID-19 physical distancing behavior

Immunocompromised adolescents showed an average moderate level of COVID-19 physical distancing intensity over the previous 4 weeks: PDS-C19 mean (SD) score was 49.1 (11.2) (*n* = 405) (Table 2). The distribution of physical distancing intensity by category among the 491 immunocompromised adolescents (full analysis set, including adolescents selected for PDS-C19 psychometric validation) was: very low, *n* = 67 (13.7%); low, *n* = 49 (10.0%); moderate, *n* = 295 (60.1%); and high/very high, *n* = 80 (16.3%). Therefore, 76.4% of immunocompromised adolescents showed moderate or high/very high physical distancing intensity.

**Table 2:** COVID-19 physical distancing and health*-*related quality of life measures of immunocompromised adolescent participants of the EAGLE study: US and UK, February to June 2023.

Measures	Total (*N* = 405)	Physical distancing level (full analysis set) (*N* = 491)			
		Very low	Low	Average	High/very high
PDS-C19	49.1 (11.2)	–	–	–	–
PedsQL™ Generic Core Scales					
Total score	58.0 (18.6)	–	–	–	–
Psychosocial health summary	58.3 (19.1)	–	–	–	–
Physical functioning	57.6 (20.7)	–	–	–	–
Emotional functioning	56.5 (20.2)	–	–	–	–
Social functioning	61.1 (21.7)	–	–	–	–
School functioning	57.2 (20.8)	–	–	–	–
DMOL					
How often do you feel lonely?	1.6 (0.8)	–	–	–	–
EQ-5D-5L					
Health utility score	0.67 (0.30)	0.88 (0.14)	0.83 (0.18)	0.63 (0.29)	0.52 (0.33)
Visual analogue scale	65.5 (19.5)	–	–	–	–
HADS					
Anxiety	8.3 (4.4)	4.7 (3.7)	5.6 (3.5)	9.2 (3.8)	10.6 (4.4)
Depression	5.7 (4.4)	1.9 (2.3)	2.4 (3.0)	6.5 (4.1)	7.5 (4.4)
WPAI-CIQ:SHP, %					
Activity impairment	30.3 (28.3)	3.1 (6.1)	8.2 (15.2)	34.2 (26.5)	54.8 (26.3)
Overall school impairment^a^	33.1 (31.8)	2.8 (7.6)	5.9 (14.9)	40.6 (31.3)	49.0 (28.7)
School absenteeism^a^	12.7 (19.0)	1.3 (4.7)	4.3 (19.1)	15.3 (19.5)	20.1 (22.3)
School presenteeism^a^	27.0 (27.4)	1.7 (4.2)	4.4 (8.5)	33.4 (27.8)	40.6 (24.1)

All data are mean (SD). En dash (–) indicates not calculated.

Total mean scores are calculated in the associations analysis set (*N* = 405), comprising the full analysis set (*n* = 491) excluding the psychometric evaluation set. Score ranges are 0–100 for PedsQL™, EQ-5D-5L visual analogue scale, and WPAI-CIQ:SHP; 0–1 for EQ-5D-5L health utility score; 0–3 for DMOL; and 0–21 for HADS.

a Only those attending classes in an academic setting (*n* = 333) completed the WPAI-CIQ:SHP overall school impairment, school absenteeism, and school presenteeism domains.

COVID-19, coronavirus disease 2019; DMOL, Direct Measure of Loneliness; HADS, Hospital Anxiety and Depression Scale; PDS-C19, Physical Distancing Scale for COVID-19 Avoidance; PedsQL™, Pediatric Quality of Life Inventory; SD, standard deviation; UK, United Kingdom; US, United States; WPAI-CIQ:SHP, Work Productivity and Activity Impairment plus Classroom Impairment Questions: Specific Health Problem.

The mean (SD) score for PDS-C19 was greater in immunocompromised adolescents than in nonimmunocompromised adolescents in our sample (49.1 [11.2] vs. 39.0 [11.5], respectively) (Supplementary Table S2), although these differences were not formally statistically tested. Among immunocompromised subgroups, those with end-stage kidney disease practiced the greatest intensity of COVID-19 physical distancing behaviors (mean [SD] score 57.9 [6.2]) (Supplementary Table S3).

### 3.3 HRQoL and other reported outcomes

In immunocompromised adolescents, the mean (SD) PedsQL™ Generic Core Scales total score was 58.0 (18.6) and psychosocial health summary score was 58.3 (19.1) (Table 2). Individual domains within this measure ranged from 56.5 (emotional functioning) to 61.1 (social functioning). The mean (SD) DMOL score for “How often do you feel lonely” (scale range, 0–3) was 1.6 (0.8).

Mean (SD) health-state utility scores among immunocompromised adolescents were 0.67 (0.30) for the EQ-5D-5L utility score and 65.5 (19.5) for the EQ-5D-5L visual analogue scale (Table 2). For EQ-5D-5L, lower utility values were observed with increasing physical distancing intensity; immunocompromised adolescents practicing high/very high physical distancing had a mean utility score of 0.52 (0.33).

The HADS mean (SD) scores for anxiety and depression among immunocompromised adolescents were 8.3 (4.4) and 5.7 (4.4), respectively (Table 2). Higher HADS scores were observed with increasing physical distancing intensity; immunocompromised adolescents practicing high/very high physical distancing had a mean (SD) anxiety score of 10.6 (4.4) and depression score of 7.5 (4.4).

For the WPAI-CIQ:SHP activity impairment domain, the mean (SD) score was 30.3 (28.3) in immunocompromised adolescents (Table 2). Among immunocompromised adolescents attending classes in an academic setting (*n* = 333), mean (SD) percent of school time missed due to avoiding COVID-19 (absenteeism) was 12.7% (19.0) and mean (SD) percent impairment while in school due to avoiding COVID-19 (presenteeism) was 27.0% (27.4). The mean (SD) score for overall school impairment was 33.1% (31.8) (Table 2). Higher levels of school absenteeism, presenteeism, and overall school impairment were observed with increasing physical distancing intensity; immunocompromised adolescents practicing high/very high physical distancing had mean (SD) values of 20.1% (22.3), 40.6% (24.1), and 49.0% (28.7), respectively.

Scores for HRQoL and other reported outcomes were in the direction of a higher degree of impairment in immunocompromised adolescents than in nonimmunocompromised adolescents in our sample (Supplementary Table S2), although these differences were not formally statistically tested. The distribution of HRQoL measures and other reported outcomes by immunocompromising category is reported in Supplementary Table S3.

### 3.4 Association between COVID-19 physical distancing and HRQoL and other measures

#### 3.4.1 Pearson correlations

**Figure 1:**
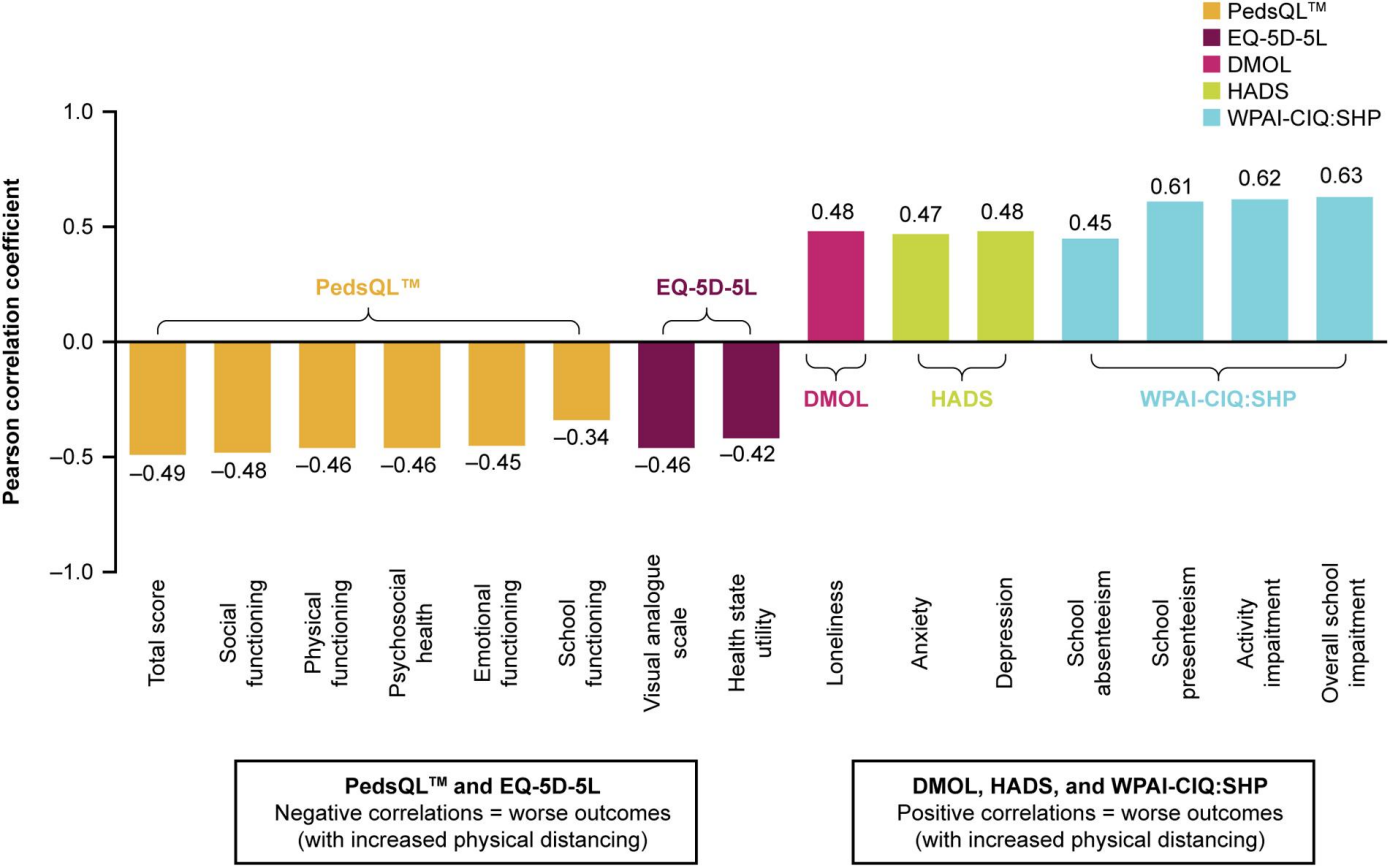
Correlations with COVID-19 physical distancing behavior intensities and health-related quality of life and other reported measures in immunocompromised adolescent participants of the EAGLE study: US and UK, February to June 2023. Pearson correlations are calculated in the associations analysis set (*N* = 405), comprising the full analysis set (*n* = 491) excluding the psychometric evaluation set. Only those attending classes in an academic setting (*n* = 333) completed the WPAI-CIQ:SHP overall school impairment, school absenteeism, and school presenteeism domains. COVID-19, coronavirus disease 2019; DMOL, Direct Measure of Loneliness; HADS, Hospital Anxiety and Depression Scale; PedsQL™, Pediatric Quality of Life Inventory; UK, United Kingdom; US, United States; WPAI-CIQ:SHP, Work Productivity and Activity Impairment plus Classroom Impairment Questions: Specific Health Problem.

The correlations between COVID-19 physical distancing intensity and HRQoL and other reported measures in immunocompromised adolescents are shown in Figure 1 and Table 3. Most correlations with PDS-C19 had an |*r*| of between 0.4 and 0.5, including most domains of the PedsQL™ Generic Core Scales, DMOL, EQ-5D-5L, HADS, and the school absenteeism domain of the WPAI-CIQ:SHP. Correlations of medium magnitude (|*r*|>0.6) were observed for the WPAI-CIQ:SHP activity impairment, school presenteeism, and overall school impairment domains. The weakest correlation (|*r*| = 0.34) observed was low in magnitude, for the school functioning domain of the PedsQL™ Generic Core Scales, while the strongest correlation (|*r*| = 0.63) observed was of medium magnitude, for the overall school impairment domain of the WPAI-CIQ:SHP.

**Table 3:** Summary of associations between PDS-C19 and health-related quality of life and other reported measures via bivariate Pearson correlations (*r*), standardized (*β*) and unstandardized (*B*) preliminary SEM regression coefficients, and *B*s from multiple univariate-outcome regression models.

Measure	Pearson correlations (*r*)^a^	SEM			Linear regression	
		*β* total^b^ (SE)	*β* direct^b^ (SE)	*B*^c^ (SE)	*B*^c^ (SE)	95% CI
PedsQL™ Generic Core Scales						
Total score	−0.49	–	–	–	−0.79 (0.11)^d^	−1.00, −0.59
Psychosocial health summary	−0.46	–	–	–	−0.77 (0.11)^d^	−0.99, −0.56
Physical functioning	−0.46	−0.49 (0.06)^d^	−0.36 (0.06)^d^	−0.67 (0.10)^d^	−0.83 (0.12)^d^	−1.07, −0.59
Emotional functioning	−0.45	−0.46 (0.06)^d^	−0.25 (0.06)^d^	−0.45 (0.10)^d^	−0.73 (0.12)^d^	−0.97, −0.49
Social functioning	−0.48	−0.49 (0.06)^d^	−0.28 (0.06)^d^	−0.56 (0.12)^d^	−0.83 (0.12)^d^	−1.07, −0.58
School functioning	−0.34	−0.42 (0.06)^d^	−0.23 (0.06)^d^	−0.43 (0.12)^d^	−0.77 (0.13)^d^	−1.01, −0.52
DMOL	0.48	0.46 (0.07)^d^	0.46 (0.07)^d^	0.05 (0.01)^d^	0.02 (0.00)^d^	0.01, 0.03
EQ-5D-5L						
Health utility score	−0.42	−0.46 (0.08)^d^	−0.26 (0.08)^d^	−0.01 (0.002)^d^	−0.01 (0.00)^d^	−0.02, −0.01
Visual analogue scale	−0.46	−0.53 (0.06)^d^	−0.39 (0.06)^d^	−0.69 (0.10)^d^	−0.71 (0.11)^d^	−0.93, −0.48
HADS						
Anxiety	0.47	0.43 (0.06)^d^	0.16 (0.06)^d^	0.06 (0.02)^d^	0.14 (0.02)^d^	0.09, 0.18
Depression	0.48	0.53 (0.06)^d^	0.24 (0.06)^d^	0.09 (0.02)^d^	0.14 (0.02)^d^	0.09, 0.18
WPAI-CIQ:SHP						
Activity impairment	0.62	0.33 (0.06)^d^	0.30 (0.06)^d^	0.76 (0.16)^d^	0.85 (0.14)^d^	0.59, 1.12
Overall school impairment^e^	0.63	–	–	–	1.00 (0.17)^d^	0.66, 1.35
School absenteeism^e^	0.45	0.23 (0.08)^d^	0.16 (0.08)	0.27 (0.14)	0.40 (0.12)^d^	0.16, 0.64
School presenteeism^e^	0.61	0.34 (0.08)^d^	0.32 (0.08)^d^	0.81 (0.20)^d^	0.94 (0.16)^d^	0.62, 1.26

Pearson correlations, SEM, and linear regressions are calculated in the associations analysis set (*N* = 405), comprising the full analysis set (*n* = 491) excluding the psychometric evaluation set. En dash (–) indicates not calculated.

a For PedsQL™ and EQ-5D-5L, negative correlations are associated with worse outcomes with higher intensities of physical distancing. For DMOL, HADS, and WPAI-CIQ:SHP, positive correlations are associated with worse outcomes with higher intensities of physical distancing.

b Standardized.

c Unstandardized.

d *P* ≤ 0.001.

e Only those attending classes in an academic setting (*n* = 333) completed the WPAI-CIQ:SHP overall school impairment, school absenteeism, and school presenteeism domains.

DMOL, Direct Measure of Loneliness; HADS, Hospital Anxiety and Depression Scale; PDS-C19, Physical Distancing Scale for COVID-19 Avoidance; PedsQL™, Pediatric Quality of Life Inventory; SE, standard error; SEM, structural equation modeling; WPAI-CIQ:SHP, Work Productivity and Activity Impairment plus Classroom Impairment Questions: Specific Health Problem.

#### 3.4.2 Preliminary SEM

Associations between PDS-C19 and the reported outcomes, as analyzed by SEM, were nominally statistically significant (*P* < 0.05; Table 3). The standardized total effect regression coefficients of PDS-C19 on the reported outcomes, as estimated using SEM, were comparable in magnitude to the corresponding bivariate correlation coefficients (Table 3), with the differences between the direct effects and correlation attributed to the role of DMOL as an intervening variable. This consistency underscores the robustness of the observed relationships, after accounting for covariates. The exception was outcomes derived from the WPAI-CIQ:SHP, where the standardized total effects were notably attenuated: activity impairment (|*r*| = 0.62 vs. *β* = 0.33), school absenteeism (|*r*| = 0.45 vs. *β* = 0.23), and school presenteeism (|*r*| = 0.61 vs. *β* = 0.34). These reductions in effect size were attributed by the models to the influence of additional contextual factors, including protective behaviors such as mask wearing (only for activity impairment) and others isolating on the adolescent's behalf, and characteristics such as household income and the presence of an immunocompromised caregiver.

#### 3.4.3 Regression

Regression models showed nominally statistically significant associations between PDS-C19 and the reported outcomes after confounder adjustment (*P*  < 0.05; Table 3). The unstandardized linear regression coefficients were of similar but slightly higher magnitude than those of the unstandardized direct effects in the SEM models (Table 3). This is primarily attributed to the use of DMOL as an intervening variable in the SEM models vs. as a covariate in the linear regression models.

## 4 Discussion

The observational, cross-sectional EAGLE study is the first study to robustly and comprehensively measure the intensity of physical distancing to avoid COVID-19 and its associations with HRQoL and related outcomes. A validated, novel physical distancing scale and several previously validated HRQoL and related instruments were used for these measures (24–26). The present analysis describes these cross-sectional outcomes among immunocompromised adolescents (aged 13–17 years) in the US and the UK, who were assessed during the transition of the COVID-19 pandemic to an endemic state (10), which was dominated by SARS-CoV-2 Omicron variants (40).

After more than 3 years since SARS-CoV-2 emergence, and 2 years since national lockdowns ended and restrictions were relaxed in the US and the UK (8, 9), more than 75% of immunocompromised US- and UK-based adolescents participating in the EAGLE study were practicing moderate or high/very high physical distancing behaviors to avoid COVID-19. Compared with previously reported reference scores among healthy populations (41–46), mean scores among immunocompromised adolescents in EAGLE were substantially lower for PedsQL™ (HRQoL) and EQ-5D-5L (health-state utilities) and were higher for HADS (anxiety and depression). In particular, the mean PedsQL™ total score of 58 reported among immunocompromised adolescents in EAGLE was around 30% lower than that reported in separate studies among US- and UK-based healthy children (aged 8–18 years; mean score, around 80) (44) and was even lower than among children (aged <18 years) with pediatric cancer (mean score, around 72) (45, 46). These findings suggest that immunocompromised adolescents in the COVID-19 post-lockdown period have worse HRQoL, worse health states, and poorer mental health than children and adolescents in the pre–COVID-19 period, including those with pediatric cancer.

More intense COVID-19 physical distancing behaviors (as measured by PDS-C19) among immunocompromised adolescents were nominally statistically significantly associated with greater impairments across all reported outcomes: HRQoL (PedsQL™), health-state utilities (EQ-5D-5L), loneliness (DMOL), anxiety and depression (HADS), and work and school impairment and productivity (WPAI-CIQ:SHP). Correlations between COVID-19 physical distancing behaviors and measured outcomes as estimated by bivariate correlation coefficients remained after accounting for potential confounding covariates in the SEM and linear regression models. The consistency of these associations across both bivariate and multivariate models highlights the strength and reliability of the observed relationships.

To date, there has been limited published evidence describing the physical distancing behaviors of immunocompromised adolescents, including those behaviors that are practiced to avoid COVID-19, and the impact these have on their daily lives (24). A previous cross-sectional study evaluated physical and mental health indicators among 355 adolescents (aged 10–18 years) with chronic conditions and 111 healthy adolescents during COVID-19 quarantine (47). In that study, female sex and fear of underlying disease activity/complication were associated with severe psychosocial dysfunction in adolescents with chronic conditions (47). Another previous survey captured the early impacts of COVID-19 on children (aged <18 years) with pediatric rheumatic diseases (23). In that survey, almost all families adopted behaviors to protect their children from COVID-19, including quarantining (96.0% of participants) and social distancing (64.9%) (23). In addition, 21.4% (75/351) of students had their school classes canceled and 9.7% (34/351) reported other changes, such as being homeschooled (23). Therefore, the findings from the EAGLE study expand our understanding of physical distancing to avoid infection in general, as well as HRQoL in immunocompromised adolescents who continued to physically distance to avoid COVID-19 for around 2 years after national lockdowns ended in the US and the UK.

Nonimmunocompromised adolescents were also included in the EAGLE study for informal benchmarking. Formal statistical testing was not conducted to confirm differences between immunocompromised and nonimmunocompromised adolescents, although descriptive statistics indicated higher mean scores for the PDS-C19 and greater impairments in all reported outcomes in immunocompromised vs. nonimmunocompromised adolescents.

Research to date has shown the association between physical distancing during the COVID-19 pandemic and worse physical and mental health among nonimmunocompromised adolescents (18–21, 48–50). A more contemporary US national survey of young adults (aged 18–29 years), including some who were adolescents during the public health emergency phase of the COVID-19 pandemic, was conducted in March 2025 and reported the long-term effects of COVID-19 pandemic restrictions among other related measures (51). Social isolation by age, which was self-reported at the time of the survey by those who were adolescents during the initial COVID-19 lockdowns in 2020, ranged from 23% to 38% (51). Furthermore, of these surveyed young adults, 55% of those who had experienced social isolation during the pandemic reported at least one symptom associated with depression, compared with only 38% of those for whom the pandemic had no lasting impact on their friendships (51).

The findings from the EAGLE study show some similarities between the immunocompromised adolescents presented here and those of the immunocompromised adults reported previously from this study (25, 26). In both populations, more intense COVID-19 physical distancing was nominally statistically significantly associated with greater HRQoL impairments, particularly as measured using WPAI-CIQ:SHP (25, 26). Interestingly, moderate or high/very high levels of physical distancing were practiced by a greater proportion (76.4%) of immunocompromised adolescents than immunocompromised adults (67.9%). As with immunocompromised adolescents, mean scores for HRQoL and health-state utilities among immunocompromised adults were lower than reference scores for the general populations (25, 26). Similar mean scores were reported for the immunocompromised adult and adolescent populations for all instruments collected in both age groups (EQ-5D-5L health-utility score and visual analogue scale, HADS anxiety and depression domains, and WPAI-CIQ:SHP activity impairment domain), although the use of age-specific instruments precludes direct comparison of other outcomes. Overall, the findings from both study populations indicate that, while physical distancing remains an important protective strategy for immunocompromised individuals, it appears to be associated with challenges in terms of HRQoL. This is an important clinical and public health consideration as COVID-19 transitions to endemicity as well as for future pandemics.

The EAGLE study has several strengths. The study included a large sample of immunocompromised adolescents, distributed across several immunocompromised categories targeted for recruitment, within the US and the UK. Reported immunocompromising conditions were validated in a randomly selected subpopulation using medical documentation, with the vast majority of self-reported immunocompromising diagnoses confirmed. Multiple study recruitment methods were used, including a virtual approach; therefore, the study was not limited to site- or clinic-based recruitment, which is very important for a vulnerable population that is physically distancing. All outcome and behavioral measures utilized were age-appropriate and validated for use, including the PDS-C19. Several of the measures also have a history of use in diverse populations, including adolescents, and published reference values are available for those instruments. Finally, the study period encompassed several months that included various SARS-CoV-2 Omicron subvariants (52).

The EAGLE study also has limitations. The design of EAGLE was cross-sectional and lacked formal comparison between immunocompromised and nonimmunocompromised participants and longitudinal observation. Therefore, causality between physical distancing behaviors to avoid COVID-19 and reported outcomes cannot necessarily be inferred from the observed associations, nor can we rule out the possibilities of reverse causation or no causation. However, interventional studies assessing whether increased physical distancing for COVID-19 avoidance causes poorer HRQoL in immunocompromised adolescents would not be feasible or ethical without effective pharmaceutical options for COVID-19 prevention. Due to the study design, we were unable to distinguish the impact of physical distancing behaviors to avoid COVID-19 on HRQoL from any impact on HRQoL caused by existing impairments from underlying immunocompromising conditions or comorbidities. The analysis revealed graded associations between physical distancing intensity to avoid COVID-19 and reported outcomes, with higher distancing scores corresponding to worse outcomes. Although associations persisted after adjustment for potential confounding, the direction and nature of this relationship cannot be definitively established in this cross-sectional analysis. The study outcomes were age-appropriate and selected so that the adolescents could self-report their outcomes; however, adolescents may have completed their self-reported outcome measures with the assistance of their caregiver if needed, which could have influenced their ability to respond truthfully. There may also be inaccuracies in reporting due to recall abilities, the time frame of reference, and the method of survey administration. The web-based recruitment system may have skewed the enrolled population towards individuals with higher technological competency and internet access via devices. Furthermore, there is potential for selection bias, as those who chose to participate may have had more severe immunocompromising conditions, worse HRQoL or worse health states, and/or had practiced more intense physical distancing behaviors to avoid COVID-19 than those who chose not to participate, and thus may have been more inclined to contribute to studies that may help lead to interventions that reduce or negate the need to physically distance in future. *P*-values were not adjusted for multiplicity across analyses; while many observed *P*-values are substantially below conventional thresholds, these results should still be interpreted in the context of the number of analyses performed. The study was conducted in two Western countries (the US and the UK) and may not be representative of populations in other countries or regions, although the study protocol has since been adapted for use in a study including non-Western countries (study in progress) and in the Netherlands (53). Finally, contemporary behaviors of individuals regarding COVID-19 may have changed since those observed during the study period.

## 5 Conclusion

The EAGLE study highlighted the prolonged burden of COVID-19 avoidance in immunocompromised adolescents, as most participants reported that, around 2 years after national lockdowns ended, they practiced moderate-to-high intensities of physical distancing behaviors to avoid COVID-19. More intense physical distancing behaviors to avoid COVID-19 were nominally statistically significantly associated with worse HRQoL and greater activity impairment in immunocompromised adolescents, with associations persisting after adjustment for potential confounding factors—these associations were generally similar to those observed in immunocompromised adults in the EAGLE study (25, 26). Despite the negative association of physical distancing intensity with HRQoL, these findings indicate that, unless effective medical interventions are available and utilized, immunocompromised individuals are likely to continue practicing physical distancing behaviors, even when COVID-19 is in an endemic phase.

## Previous presentation

Adolescent data from the EAGLE study were presented at the annual Congress of the European Society of Paediatric Infectious Diseases, May 26–30, 2025, Bucharest, Romania (Poster; Abstract #733).

## Data Availability

Data underlying the findings described in this manuscript may be obtained in accordance with AstraZeneca's data sharing policy described at: https://www.astrazenecaclinicaltrials.com/our-transparency-commitments/. Data for studies directly listed on Vivli can be requested through Vivli at www.vivli.org. Data for studies not listed on Vivli could be requested through Vivli at https://vivli.org/members/enquiries-about-studies-not-listed-on-the-vivli-platform/. The AstraZeneca Vivli member page is also available outlining further details: https://vivli.org/ourmember/astrazeneca/. Further enquiries can be made to the corresponding author.
