# Supplementary material for: Assessing the association of physical distancing to avoid COVID-19 with health-related quality of life in immunocompromised adolescents: results from the cross-sectional observational EAGLE study
Source: Front Pediatr. 2026 May 28;14:1771834. doi: 10.3389/fped.2026.1771834 (PMC13253539; doi:10.3389/fped.2026.1771834)
Supplement: Supplementary file 1 [file Supplementaryfile1.pdf]

Assessing the association of physical distancing to avoid COVID-19 with health-related quality of life in immunocompromised adolescents: results from the cross-sectional observational EAGLE study

Paul Williams[1#], Timothy A. Herring[2#*], Renata T. C. Yokota[3], Sudhir Venkatesan[4], Klas Bergenheim[5], Johan L. Severens[6], Philip A. Powell[7], James C. Marcus[8], Stephanie Philpott[9], Sara Bestea[10], Jeffrey M. Rohay[11#], Tiago Maia[12#], Sylvia Taylor[13#], Marieke Krol[14], and James W. Varni[15]

[1]Medical Evidence, BioPharmaceuticals Medical, AstraZeneca, Gothenburg, Sweden

[2]Medical Evidence, Vaccines & Immune Therapies, BioPharmaceuticals Medical, AstraZeneca, Wilmington, DE, United States

[3]P95 Clinical and Epidemiology Services, Leuven, Belgium

[4]BPM Evidence Statistics, BioPharmaceuticals Medical, AstraZeneca, Cambridge, United Kingdom

[5]Health Economics & Payer Evidence, BioPharmaceuticals Market Access, AstraZeneca, Gothenburg, Sweden

[6]Severens HTA Consultancy, Heemskerk, Netherlands

[7]Philip A Powell Consulting, Sheffield, United Kingdom

[8]IQVIA, Washington, DC, United States

[9]IQVIA, Reading, United Kingdom

[10]IQVIA, Madrid, Spain

[11]IQVIA, New York, NY, United States

[12]IQVIA, Porto Salvo, Portugal

[13]Medical Evidence, Vaccines & Immune Therapies, BioPharmaceuticals Medical, AstraZeneca, Cambridge, United Kingdom

[14]IQVIA, Amsterdam, Netherlands

[15]Texas A&M University, College Station, TX, United States

[#]At the time of this study

*Corresponding author

Supplementary Material

Supplementary Data

Eligibility criteria

Inclusion criteria for pediatric participants (aged <18 years):

- Aged 6 months to 17 years (only adolescents, who were aged 13–17 years, were included in this analysis)
- Resided in the United States (US) or the United Kingdom (UK)
- Able to understand English or Spanish (for US-based participants only) at a level typical of their age, with the assistance of their caregiver, if needed
- Willing and able to complete questionnaires about their feelings and health, and simple descriptive questions lasting less than 15 min, with the assistance of their caregiver, if needed
- Willing and able to provide assent to participate in the study
- Have a formal caregiver who meets the caregiver eligibility criteria

Exclusion criteria:

- Current participation in a clinical trial for experimental/investigational treatments for immunocompromising conditions and/or preventions or treatments of severe acute respiratory syndrome coronavirus 2 infection and/or coronavirus disease 2019 (COVID-19)
- Current hospitalization or admission to an inpatient facility
- History of AZD7442 (tixagevimab/cilgavimab) administration or any other passive immunization therapy for COVID-19 (history of vaccination against COVID-19 was acceptable)

Reported outcome questionnaires

- The **Pediatric Quality of Life Inventory™ (PedsQL™)** Generic Core Scales (1) (teen report for ages 13–18 years) is a 23-item instrument divided into four domains: physical functioning, emotional functioning, social functioning, and school functioning. Each item has five ordinal response options (“Never,” “Almost never,” “Sometimes,” “Often,” and “Almost always”), which were reverse-scored and linearly transformed onto a 0–100 scale, with higher scores indicating better health-related quality of life.
- The **Direct Measure of Loneliness (DMOL)** (2) is a single-item measure assessing current feelings of loneliness (“How often do you feel lonely?”), with five ordinal responses (“Often or always,” “Some of the time,” “Occasionally,” “Hardly ever,” and “Never”). Due to limited

distinction between “Some of the time” and “Occasionally,” these were combined for analysis. Final categories were coded as “Never” (0), “Hardly ever” (1), “Some of the time/Occasionally” (2), and “Often or always” (3). For inferential analyses, recoded DMOL was treated as an interval variable ranging from 0–3.

- The **EQ-5D-5L** questionnaire (3) is a standard measure of health utility on the day of administration. The tool consists of two components, a descriptive system and a visual analogue scale. When the descriptive system is linked to a value set, utility scores can be derived for mobility, self-care, usual activities, pain/discomfort, and anxiety/depression, each with five levels (“No problem,” “Slight problems,” “Moderate problems,” “Severe problems,” and “Extreme problems”). Responses yield a health state which is converted into a utility score of 0–1, with higher scores indicating better health-related quality of life. The visual analogue scale records the participants’ health on a 0–100 scale, with higher scores indicating better health.

- The **Hospital Anxiety and Depression Scale (HADS)** (4) comprises an anxiety scale and a depression scale which assess mental health over the previous week. Each scale consists of seven items scored on a 0–3 scale, resulting in a total score of 0–21. Scores of <7 indicate non-cases; 8–10, mild cases; 11–14, moderate cases; and 15–21, severe cases.

- The **Work Productivity and Activity Impairment plus Classroom Impairment Questions: Specific Health Problem (WPAI-CIQ:SHP)** (5) is a version of the Work Productivity and Activity Impairment questionnaire that includes specific questions for classroom impairment and specific health problems. The questionnaire has 10 items scored on a scale of 0–10, with 0 indicating no effect and 10 indicating that the exposure completely prevented the individual from working or performing in school (5). The WPAI-CIQ:SHP scores on school absenteeism (proportion of school hours missed), school presenteeism (degree that health affected productivity while in school or attending classes), overall work or school impairment (linear combination of absenteeism and presenteeism), and activity impairment (degree to which health affected regular activities). Scores are expressed as impairment percentages, with higher percentages indicating greater impairment and less productivity.

Supplementary Figure S1. Illustration, using HRQoL outcomes, of the general path schema employed in SEM models

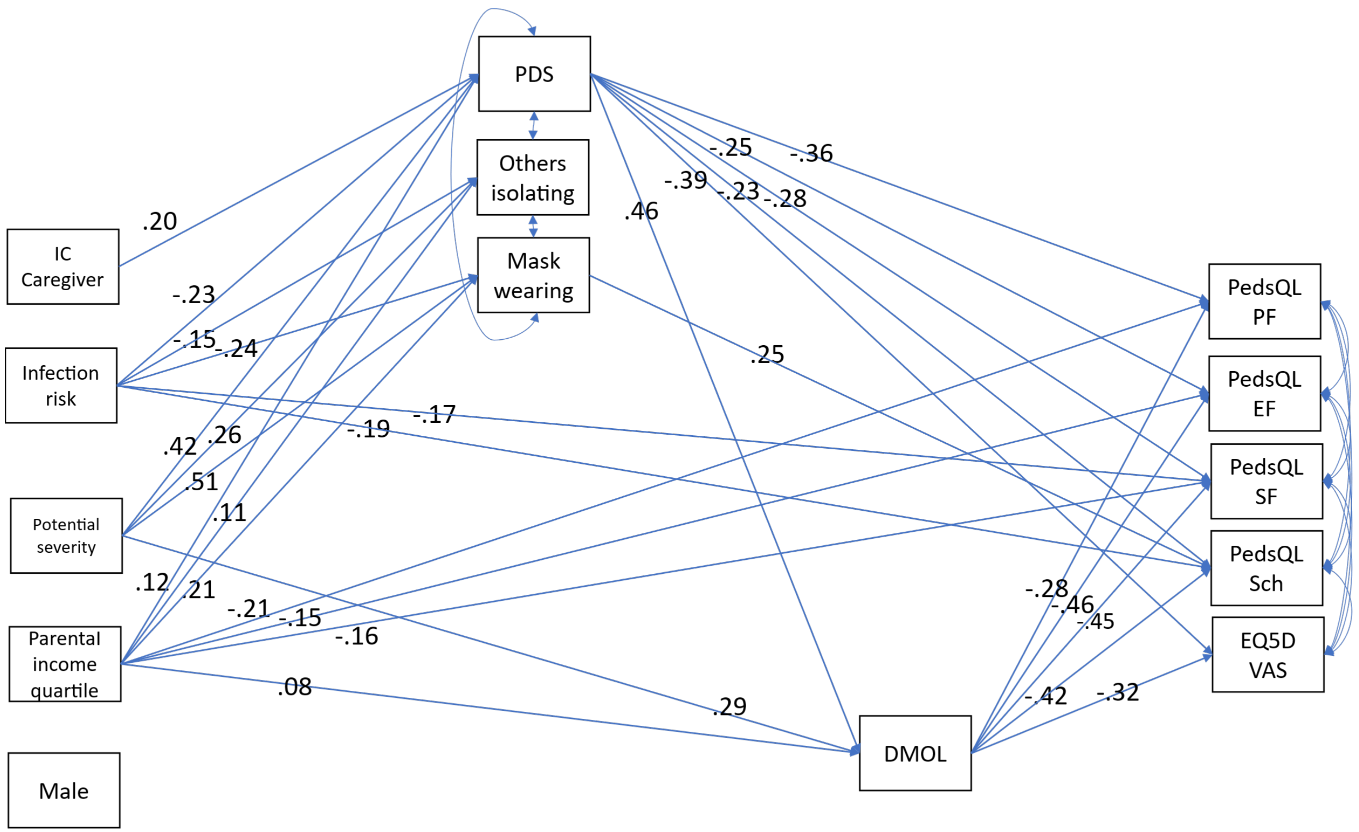

The diagram represents all direct and indirect paths between the explanatory variables (left-most side) and the outcomes of interest (right-most side; in this example, PedsQL™ domains [PF, EF, SF, and Sch] and EQ-5D VAS as HRQoL outcomes for immunocompromised adolescents) and mediating variables (middle). EF, emotional functioning; DMOL, Direct Measure of Loneliness; HRQoL, health-related quality of life; IC, immunocompromised; PedsQL™, Pediatric Quality of Life Inventory; PDS, Physical Distancing Scale for COVID-19 Avoidance; PF, physical functioning; Sch, school functioning; SEM, structural equation modeling; SF, social functioning; VAS, visual analogue scale.

Supplementary Figure S2. Flow chart of participant screening and inclusion across the US and UK

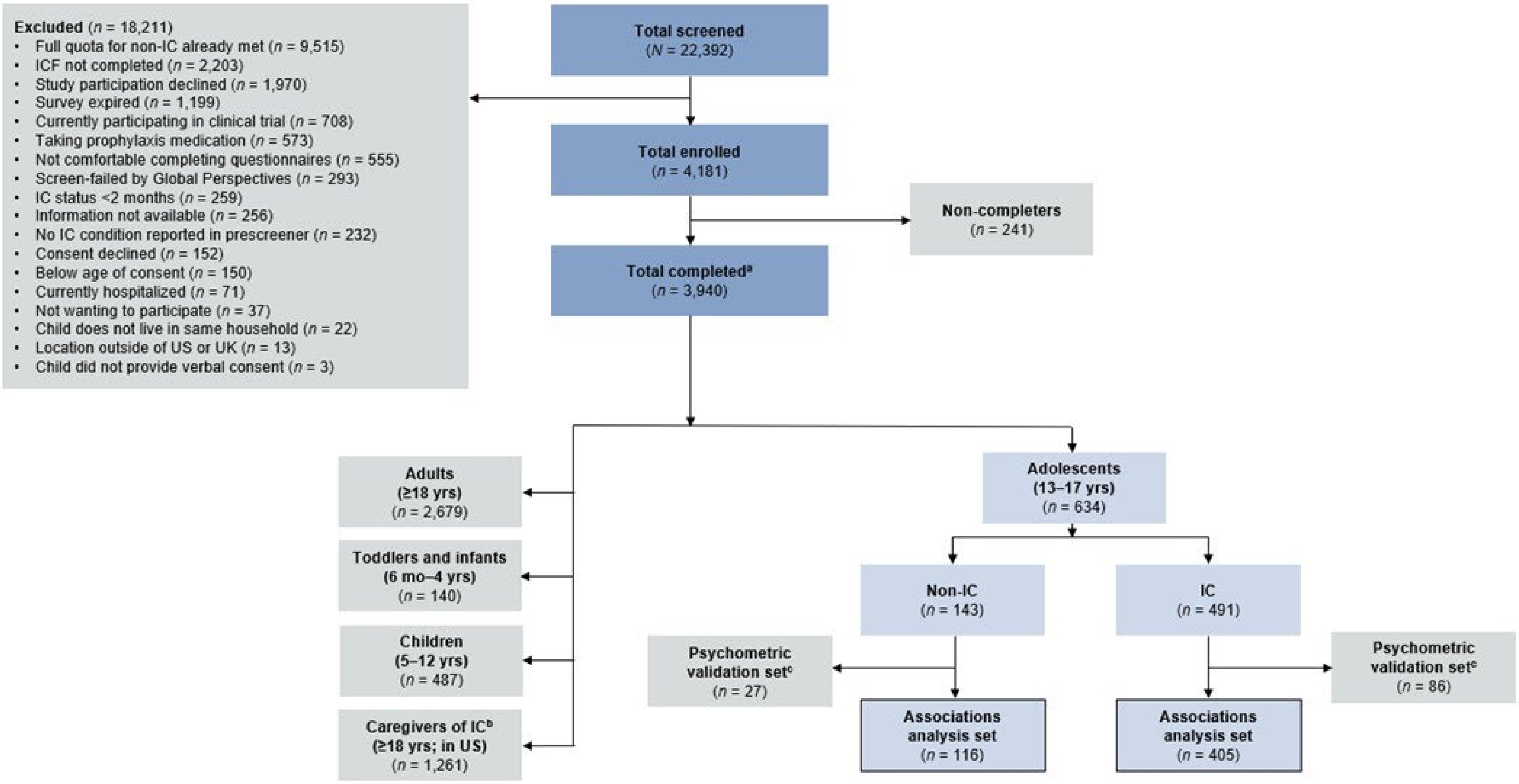

[a]Participants who provided fully completed surveys. [b]Caregiver and child dyads were counted together as a single unit, as they completed one survey together. [c]Participants who completed the survey through the PDS-C19 were eligible for inclusion in the psychometric validation set even if they did not complete the full survey. Accordingly, the psychometric validation set (*n* = 152 adolescents) also includes *n* = 15 IC and *n* = 24 non-IC adolescents who did not complete the full survey and were not part of the FAS. FAS, full analysis set; IC, immunocompromised; ICF, informed consent form; mo, months; PDS-C19, Physical Distancing Scale for COVID-19 Avoidance; UK, United Kingdom; US, United States; yrs, years.

Supplementary Table S1. Demographic characteristics of the nonimmunocompromised adolescent participants of the EAGLE study: US and UK, February to June 2023

Characteristic, *n* (%)	US (*n* = 51)	UK (*n* = 92)	Total (*N* = 143)
Sex			
Female	22 (43.1)	43 (46.7)	65 (45.5)
Race (US)[a]			
White	45 (88.2)	–	–
Black or African American	7 (13.7)	–	–
Hispanic	4 (7.8)	–	–
Asian	0	–	–
American Indian or Alaska Native	0	–	–
Native Hawaiian or other Pacific Islander	0	–	–
Other	0	–	–
Prefer not to say	0	–	–
Race (UK)[a]			
White	–	82 (89.1)	–
Black, Black British, Caribbean, or African	–	2 (2.2)	–

**Characteristic, *n* (%)**	**US (*n* = 51)**	**UK (*n* = 92)**	**Total (*N* = 143)**
Mixed or multiple ethnic groups	–	4 (4.3)	–
Asian or Asian British	–	4 (4.3)	–
Prefer not to say	–	0	–
Adults (aged ≥18 years) living in household			
0	0	1 (1.1)	1 (0.7)
1	6 (11.8)	11 (12.0)	17 (11.9)
2	33 (64.7)	55 (59.8)	88 (61.5)
≥3	12 (23.5)	25 (27.2)	37 (25.9)
Children (aged <18 years) living in household			
0	0	5 (5.4)	5 (3.5)
1	29 (56.9)	47 (51.1)	76 (53.1)
2	17 (33.3)	33 (35.9)	50 (35.0)
≥3	5 (9.8)	7 (7.6)	12 (8.4)
Attending classes in an academic setting[b]			
Yes	25 (65.8)	66 (84.6)	91 (78.4)

**Characteristic, *n* (%)**	**US (*n* = 51)**	**UK (*n* = 92)**	**Total (*N* = 143)**
No	13 (34.2)	12 (15.4)	25 (21.6)

Data are from the full analysis set (*N* = 143), except "attending classes in an academic setting" data, which are from the associations analysis set (*N* = 116). En dash (–) indicates not applicable.

[a]Participants may have selected more than one.

[b]Middle school, high school, college, graduate school, additional course work, etc.

UK, United Kingdom; US, United States.

Supplementary Table S2. COVID-19 physical distancing and HRQoL measures of immunocompromised and nonimmunocompromised adolescent participants of the EAGLE study: US and UK, February to June 2023

Measures	Immunocompromised, total (*N* = 405)	Nonimmunocompromised				
		Total (*N* = 116)	Physical distancing level (full analysis set; *N* = 143)			
			Very low	Low	Average	High/ Very high
PDS-C19	49.1 (11.2)	39.0 (11.5)	–	–	–	–
PedsQL™ Generic Core Scales						
Total score	58.0 (18.6)	79.7 (16.5)	–	–	–	–
Psychosocial health summary	58.3 (19.1)	76.2 (18.7)	–	–	–	–
Physical functioning	57.6 (20.7)	86.5 (16.8)	–	–	–	–
Emotional functioning	56.5 (20.2)	71.9 (21.6)	–	–	–	–

Measures	Immunocompromised, total (*N* = 405)	Nonimmunocompromised				
		Total (*N* = 116)	Physical distancing level (full analysis set; *N* = 143)			
			Very low	Low	Average	High/ Very high
Social functioning	61.1 (21.7)	79.2 (20.3)	–	–	–	–
School functioning	57.2 (20.8)	77.3 (20.2)	–	–	–	–
DMOL						
How often do you feel lonely?	1.6 (0.8)	1.3 (0.9)	–	–	–	–
EQ-5D-5L						
Health utility score	0.67 (0.23)	0.85 (0.18)	0.90 (0.13)	0.84 (0.21)	0.85 (0.16)	0.73 (0.34)
Visual analogue scale	65.5 (19.5)	77.3 (19.1)	–	–	–	–

Measures	Immunocompromised, total (*N* = 405)	Nonimmunocompromised				
		Total (*N* = 116)	Physical distancing level (full analysis set; *N* = 143)			
			Very low	Low	Average	High/ Very high
HADS						
Anxiety	8.3 (4.4)	6.0 (4.7)	4.8 (4.2)	5.2 (4.6)	7.2 (4.5)	7.1 (5.2)
Depression	5.7 (4.4)	3.7 (4.1)	2.4 (3.7)	2.8 (3.5)	5.3 (4.0)	5.5 (5.8)
WPAI-CIQ:SHP, %						
Activity impairment	30.3 (28.3)	18.7 (27.5)	4.1 (11.1)	15.2 (22.9)	33.7 (29.0)	41.0 (37.6)
Overall school impairment[a]	33.1 (31.8)	18.6 (29.0)	4.8 (11.1)	18.1 (31.7)	34.0 (31.9)	33.3 (41.3)
School absenteeism[a]	12.7 (19.0)	2.9 (11.7)	0.0 (0.0)	8.3 (23.1)	4.8 (13.5)	0.0 (0.0)

**Measures**	**Immunocompromised, total ($N = 405$)**	**Nonimmunocompromised**				
		**Total ($N = 116$)**	**Physical distancing level (full analysis set; $N = 143$)**			
			**Very low**	**Low**	**Average**	**High/ Very high**
School presenteeism[a]	27.0 (27.4)	17.3 (27.5)	4.8 (11.1)	15.8 (28.4)	31.8 (31.2)	33.3 (41.3)

All data are mean (SD). En dash (–) indicates not calculated. Total mean scores are calculated in the associations analysis set, comprising the full analysis set excluding the psychometric evaluation set. Score ranges are 0–100 for PedsQL™, EQ-5D-5L visual analogue scale, and WPAI-CIQ:SHP; 0–1 for EQ-5D-5L health utility score; 0–3 for DMOL; and 0–21 for HADS. Formal statistical testing was not conducted to confirm differences between immunocompromised and nonimmunocompromised adolescents.

[a]For nonimmunocompromised adolescents attending classes in an academic setting, 89 in the associations analysis set completed the school presenteeism and overall school impairment domains, 90 completed the school absenteeism domain, and all 116 completed the activity impairment domain. For immunocompromised individuals, 333 in the associations analysis set completed the WPAI-CIQ:SHP overall school impairment, school absenteeism, and school presenteeism domains.

COVID-19, coronavirus disease 2019; DMOL, Direct Measure of Loneliness; HADS, Hospital Anxiety and Depression Scale; HRQoL, health-related quality of life; PDS-C19, Physical Distancing Scale for COVID-19 Avoidance; PedsQL, Pediatric Quality of Life Inventory; SD, standard deviation; UK, United Kingdom; US, United States; WPAI-CIQ:SHP, Work Productivity and Activity Impairment plus Classroom Impairment Questions: Specific Health Problem.

Supplementary Table S3. Distribution of COVID-19 physical distancing intensity and HRQoL measures by immunocompromising category in immunocompromised adolescent participants of the EAGLE study: US and UK, February to June 2023

**Measures**	**Primary immuno-deficiency disorder (*n* = 103)**	**Solid organ or stem cell transplant (*n* = 87)**	**Immuno-suppressant treatment (*n* = 42)**	**Blood cancer (*n* = 24)**	**COVID-19 vaccine contra-indication (*n* = 21)**	**End-stage chronic kidney disease (*n* = 13)**	**Solid tumors, on active treatment (*n* = 8)**	**Uncontrolled HIV infection (*n* = 7)**	**Other disorder (*n* = 100)**
PDS-C19	52.6 (10.4)	43.6 (10.7)	42.7 (12.0)	49.0 (10.7)	51.7 (10.5)	57.9 (6.2)	55.1 (5.1)	56.5 (6.9)	50.3 (10.1)
PedsQL™ Generic Core Scales									
Total score	54.1 (19.7)	60.9 (15.3)	69.5 (17.8)	55.9 (19.1)	63.4 (14.4)	50.3 (16.6)	45.4 (19.6)	27.0 (7.5)	58.3 (17.7)
Psychosocial health summary	55.1 (20.8)	61.5 (15.0)	70.2 (16.6)	56.6 (20.0)	61.9 (14.4)	52.4 (14.4)	44.6 (19.9)	23.8 (10.0)	57.8 (18.6)
Physical functioning	52.4 (20.3)	60.0 (18.0)	68.2 (23.4)	54.6 (19.7)	66.1 (18.3)	46.4 (22.2)	46.9 (20.5)	33.0 (5.4)	59.4 (20.0)
Emotional functioning	53.0 (21.5)	59.7 (15.6)	67.9 (19.0)	58.3 (18.7)	59.5 (17.5)	45.8 (17.9)	48.1 (18.7)	21.4 (10.3)	56.3 (20.5)

**Measures**	**Primary immuno-deficiency disorder (*n* = 103)**	**Solid organ or stem cell transplant (*n* = 87)**	**Immuno-suppressant treatment (*n* = 42)**	**Blood cancer (*n* = 24)**	**COVID-19 vaccine contra-indication (*n* = 21)**	**End-stage chronic kidney disease (*n* = 13)**	**Solid tumors, on active treatment (*n* = 8)**	**Uncontrolled HIV infection (*n* = 7)**	**Other disorder (*n* = 100)**
Social functioning	56.2 (23.5)	64.3 (17.9)	75.4 (18.6)	56.9 (21.7)	65.5 (17.7)	53.1 (16.7)	45.0 (23.3)	28.6 (9.0)	62.2 (21.0)
School functioning	56.0 (22.1)	60.5 (16.0)	67.4 (17.7)	54.6 (25.0)	60.7 (17.8)	58.5 (15.2)	40.6 (20.6)	21.4 (15.7)	54.8 (21.2)
DMOL									
How often do you feel lonely?	1.8 (0.7)	1.2 (0.9)	1.3 (1.0)	1.7 (0.8)	1.9 (0.5)	1.9 (0.5)	2.0 (0.0)	2.1 (0.4)	1.7 (0.7)
EQ-5D-5L									
Health utility score	0.60 (0.30)	0.73 (0.25)	0.79 (0.25)	0.54 (0.38)	0.81 (0.22)	0.58 (0.28)	0.62 (0.14)	0.02 (0.29)	0.69 (0.28)
Visual analogue scale	62.7 (19.5)	66.9 (18.9)	73.3 (18.5)	59.7 (20.1)	69.8 (16.9)	51.6 (14.7)	57.5 (21.9)	34.7 (28.6)	68.9 (17.2)

Measures	Primary immuno-deficiency disorder (*n* = 103)	Solid organ or stem cell transplant (*n* = 87)	Immuno-suppressant treatment (*n* = 42)	Blood cancer (*n* = 24)	COVID-19 vaccine contra-indication (*n* = 21)	End-stage chronic kidney disease (*n* = 13)	Solid tumors, on active treatment (*n* = 8)	Uncontrolled HIV infection (*n* = 7)	Other disorder (*n* = 100)
HADS									
Anxiety	8.8 (4.9)	6.8 (4.1)	7.3 (5.4)	8.0 (3.3)	8.5 (3.1)	9.9 (2.7)	11.5 (3.1)	11.6 (2.3)	9.0 (4.1)
Depression	5.5 (4.6)	4.7 (4.4)	4.6 (4.3)	6.9 (4.3)	6.3 (4.3)	8.1 (2.6)	10.5 (3.8)	11.0 (2.2)	5.9 (4.0)
WPAI-CIQ:SHP, %									
Activity impairment	45.2 (26.1)	17.5 (25.5)	15.5 (23.0)	27.9 (30.4)	37.6 (32.5)	42.3 (32.2)	43.8 (25.0)	55.7 (19.0)	26.9 (24.3)
Overall school impairment[a]	54.0 (28.3)	17.5 (28.5)	19.1 (26.7)	20.8 (24.6)	30.3 (32.3)	50.3 (30.6)	37.6 (29.0)	71.5 (25.2)	30.9 (28.9)
School absenteeism[a]	22.9 (19.9)	7.6 (18.0)	8.2 (16.7)	9.0 (15.7)	12.1 (19.2)	19.3 (23.8)	17.6 (26.4)	40.0 (20.5)	7.5 (13.5)
School presenteeism[a]	43.6 (25.3)	13.9 (23.4)	13.8 (20.3)	14.4 (20.4)	24.7 (29.2)	43.0 (25.8)	28.0 (16.4)	55.0 (31.5)	27.6 (26.5)

All data are mean (SD). Total mean scores are calculated in the associations analysis set ($N = 405$), comprising the full analysis set ($n = 491$) excluding the psychometric evaluation set. Score ranges are 0–100 for PedsQL™, EQ-5D-5L visual analogue scale, and WPAI-CIQ:SHP; 0–1 for EQ-5D-5L health utility score; 0–3 for DMOL; and 0–21 for HADS.

[a]Only those attending classes in an academic setting completed the WPAI-CIQ:SHP overall school impairment, school absenteeism, and school presenteeism domains.

COVID-19, coronavirus disease 2019; DMOL, Direct Measure of Loneliness; HADS, Hospital Anxiety and Depression Scale; HRQoL, health-related quality of life; PDS-C19, Physical Distancing Scale for COVID-19 Avoidance; PedsQL, Pediatric Quality of Life Inventory; SD, standard deviation; UK, United Kingdom; US, United States; WPAI-CIQ:SHP, Work Productivity and Activity Impairment plus Classroom Impairment Questions: Specific Health Problem.

References

1. Varni JW, Seid M, Kurtin PS. PedsQL 4.0: reliability and validity of the Pediatric Quality of Life Inventory version 4.0 generic core scales in healthy and patient populations. *Med Care* (2001) 39(8):800-12. doi: 10.1097/00005650-200108000-00006.
2. El-Osta A, Alaa A, Webber I, Riboli Sasco E, Bagkeris E, Millar H, et al. How is the COVID-19 lockdown impacting the mental health of parents of school-age children in the UK? A cross-sectional online survey. *BMJ Open* (2021) 11(5):e043397. doi: 10.1136/bmjopen-2020-043397.
3. Herdman M, Gudex C, Lloyd A, Janssen M, Kind P, Parkin D, et al. Development and preliminary testing of the new five-level version of EQ-5D (EQ-5D-5L). *Qual Life Res* (2011) 20(10):1727-36. doi: 10.1007/s11136-011-9903-x.
4. Zigmond AS, Snaith RP. The hospital anxiety and depression scale. *Acta Psychiatr Scand* (1983) 67(6):361-70. doi: 10.1111/j.1600-0447.1983.tb09716.x.
5. Reilly MC, Zbrozek AS, Dukes EM. The validity and reproducibility of a work productivity and activity impairment instrument. *Pharmacoeconomics* (1993) 4(5):353-65. doi: 10.2165/00019053-199304050-00006.
